# How social and media cues induce live streaming impulse buying? SOR model perspective

**DOI:** 10.3389/fpsyg.2024.1379992

**Published:** 2024-05-15

**Authors:** Yu Xiang Xia, Seong Wook Chae, Yi Cai Xiang

**Affiliations:** ^1^Department of Economics, Jiujiang University, Jiujiang, China; ^2^Jiangxi Open Economy Research Center, Jiujiang, China; ^3^Department of Digital Business, Hoseo University, Cheonan, Republic of Korea

**Keywords:** live streaming e-commerce, social cues, media cues, emotion, perceived uncertainty, urge to buy impulsively

## Abstract

Live streaming is revolutionizing the landscape of e-commerce, creating new opportunities for platforms and e-tailers to improve their performance. However, little is known about the underlying mechanisms that shape consumer behavior in this burgeoning business phenomenon. This study aims to shed light on the relationships between environmental cues generated by live streaming and online impulse buying. Drawing upon the Stimulus-Organism-Response framework, a comprehensive model was formulated to explore how social cues (streamer interaction, peer interaction) and media cues (vividness, realness) impact pleasure, arousal, perceived uncertainty, and subsequently induce consumers’ urge to buy impulsively. The model was tested by survey data from 403 consumers. SPSS and PLS are employed to verify the model. The findings revealed that realness and streamer interaction can reduce perceived uncertainty and foster a pleasant consumer experience, while vividness and peer interaction serve to awaken and delight consumers. Pleasure, arousal, and perceived uncertainty mediate antecedent variables’ effects on urge to buy impulsively in a parallel and reverse way, and emotions exert a more powerful influence. This study enriched the research on the influence mechanisms of impulse buying driven by live streaming and provided suggestions for platforms and streamers to optimize product display and guide interaction, which is conducive to leveraging the advantages of live streaming and creating greater commercial value.

## 1 Introduction

Live streaming is an innovative medium that enables online merchants to provide consumers with a personalized experience through video presentation and real-time interaction (Yan et al., 2023). It caters to the digital-savvy generation’s expectations for in-depth interaction and rich media information and has rapidly evolved into a global trend. In 2016, Taobao pioneered the integration of live streaming functionality. Afterward, the user activity (Su, 2019) and the conversion rate (Wang et al., 2018) improved significantly. Depending on these advantages, live streaming e-commerce (LSE) attracted a mass of users, shown huge market potential (iResearch, 2022), and successfully aroused a wide range of interest from marketers, retailers, and platforms. Especially, rely on the COVID-19 hurricane, the penetration rate of LSE has surged even further. However, converting traffic into effective purchases remains a challenge. For instance, on February 14, 2023, the Durex studio attracted more than 3 million viewers, yet the gross merchandise volume was less than CNY 6000. Many e-commerce retailers are playing the role of live streaming “companion runners.” Understanding how to effectively leverage these advantages to support consumers’ quick decision-making is crucial for “traffic monetization.” Previous studies have investigated this question from various angles, including IT affordance (Putra and Hayadi, 2024), various presence (Ming et al., 2021; Zhang et al., 2022b), and ZhongYong Tendency (Gao et al., 2022), etc. These studies have unlocked the power of technical features, novel shopping experiences, and individual traits on live streaming impulsive consumption. However, it still necessary to further clarify the essential characteristics of LSE environment and the psychological mechanisms that induce impulse buying.

Environmental cues are those elements that can be designed by marketers to stimulate consumers’ perceptions (Wang D. et al., 2022). When consumers do not have a clear purchase goal, environmental cues exert an even more important role than the product itself (Bohl, 2012). This assumption is consistent with most live streaming consumption scenarios. Until consumers access the live studio, they do not know what products the streamer is recommending. The streamer-viewer and viewer-viewer interaction co-determines the direction of the live content. This novel experience is definitely different from solo, self-searching shopping in traditional e-commerce. Besides, live streaming transcends the limitations of static, dull product presentations. It dynamically displays product details through real-time video and integrates the background, streamer, and product to create a more realistic scene. Wang D. et al. (2022) argued that the definitions and dimensions of atmospheric cues varied with the context-specific properties. They argued that visibility and instant interaction were the most significant atmospheric cue characteristics in LSE. Based on the above, we conceptualized the unique environmental cues created by LSE as media cues and social cues, and further decomposed them into vividness vs. realness, streamer interaction vs. peer interaction.

Moreover, live streaming is a hedonic information system (Ming et al., 2021). Consumers watch live streaming not only for shopping but also for pleasure (Ma et al., 2022). Especially after COVID-19, consumers who have experienced loneliness and boredom expect emotional support from it. Thus, it’s crucial to pay attention to their emotional changes. In this study, pleasure and arousal are adopted to measure personal emotional state. Furthermore, Wang X. et al. (2022) argued that subjective factors such as emotion and perception jointly determined consumers’ internal state. Shi et al. (2018) claimed that perceived uncertainty was a major barrier to online transactions. In LSE, the main objects are experience products^1^ (China Consumers Association, 2020). The inherent nature of these products exacerbates the difficulty of accurately predicting transaction outcomes. Previous studies have emphasized the importance of perceived uncertainty, however, Chen et al. (2019) merely provided a conceptual model. Zhang et al. (2019) and Guo et al. (2021) defined the antecedent variable of perceived uncertainty as a holistic concept—“live streaming strategy” or “cross-border e-commerce live streaming features,” which could not provide a more detailed understanding. Given the above reasons, we employ a Stimulus-Organism-Response (SOR) framework that attempts to address the following question:

RQ: How do social and media cues induce impulse buying in LSE through consumers’ emotions and cognition?

The contribution of this study is manifested in two folds. Firstly, different from previous studies, by comparing with traditional e-commerce, this study defines social cues and media cues as stimuli and explores their different effects on organisms, thus providing a more in-depth explanation of the formation mechanism of LSE impulse buying. Secondly, based on the emotion-cognitive framework, this study employs personal emotions and perception as mediators, revealing the mental processes from external stimuli to behavioral responses. Through this research, more detailed suggestions are drawn for platforms and streamers in optimizing product display and guiding interaction, which is conducive to fostering a preferable shopping environment and creating greater commercial value.

## 2 Literature review and theoretical background

### 2.1 Previous studies on live streaming shopping

Live streaming commerce (LSC) refers to a marketing model in which streamers provide product information and product trials to consumers through interpersonal communication relying on live streaming, thus promoting consumers’ purchase intention (Guo et al., 2021). It can be realized in two ways, including e-commerce websites integrated live-streaming functions or embedding commercial activities on live streaming platforms (Wongkitrungrueng et al., 2020). Given e-commerce platforms are characterized by high consumer concentration, live streaming shopping predominantly takes place on e-commerce platforms.

Previous studies have discussed the commercial impact of live streaming environmental cues. These features primarily fell into two categories: social factors and technical factors. These social factors are represented by Wanghong attractiveness or Wanghong trustworthiness (Park and Lin, 2020), positive evaluations towards the vendor based on their reputation, hedonic effort, price advantage, etc., as well as a crowd perception of other buyers (Leeraphong and Sukrat, 2018). It may also derive from a relationship state with the streamer, such as para-social interactions (Ko and Chen, 2020), affective intensives (Zhang et al., 2022a), swift guanxi (Men and Zheng, 2019), etc. The second stream focuses on the technical features of live streaming, including technology enablers such as visibility affordance, meta-voicing affordance, and guidance shopping affordance (Sun et al., 2019); Learning-related affordances like coactive vicarious learning and independent vicarious learning (Li et al., 2020); And IT-mediated mechanisms such as value transmission, vicarious experience learning, and product presentation (Chen et al., 2019). Recent research tends to integrate social and technological drivers. Zhang M. et al. (2022) argued that a single technical or social perspective is insufficient to evaluate the performance of an information system. From a socio-technical perspective, they proposed that social enablers and technical enables synergistically determined consumers’ attitudes towards streamers and products. Zheng et al. (2023) constructed a stream-streamer-viewer framework, advocating that the social presence and interactivity enabled by the stream, along with the attractiveness and expertise of the streamer, stimulated flow experience and led to continuous watching and purchase intention.

Previous research has yielded valuable insights for us. However, social influence is often interpreted as the presence and the number of other viewers (Leeraphong and Sukrat, 2018; Ko and Chen, 2020; Zhang et al., 2022b), the features of streamers (Wang et al., 2018; Hou et al., 2019; Ko and Chen, 2020; Park and Lin, 2020; Xu et al., 2020a; Zheng et al., 2023), and the action and enthusiasm of sellers (Leeraphong and Sukrat, 2018). Besides, as for the internal mechanism of environmental cues inducing behavior, previous research has emphasized the mediating role of relationship status, flow experience (Dong et al., 2023), personal characteristics (Gao et al., 2022), and community-triggered emotions (Lim et al., 2020; Li et al., 2021), few studies have focused on personal emotional changes (Xu et al., 2023) and emotional subdivided dimensions. Moreover, while the uncertainty concern surrounding experience products is a critical barrier to online consumption, few studies have offered concrete suggestions on how to eliminate it. Given these considerations, this study attempts to integrate two typical social cues and media properties, as well as personal emotions and perceptions, into the interpretation of impulse buying. Exploring their specific influence mechanism and comparing their differences in efficacy is essential and interesting.

### 2.2 Theoretical background-SOR

The SOR model was proposed by Mehrabian and Russell (1974), which denied the direct effect of stimulus on behavior in the “stimulus–response” (SR) model (Watson, 1913), and maintained that environmental stimulus leads to approach or avoidance behavior through the internal state of the individual. In subsequent studies, scholars tried to categorize these stimuli, e.g., high/low load environments (Mehrabian and Russell, 1974), high/low task-relevant environmental cues (Eroglu et al., 2003), as well as marketing stimuli, platform stimuli, and other stimuli (Xu et al., 2023). Regarding the internal state, Russell and Pratt (1980) initially interpreted it as an emotional state. Watson and Spence (2007) divided research on marketing emotion into three stages: the categories approach, the dimensions approach, and the cognitive appraisal approach. They advocated that the cognitive appraisal approach provides a more complete framework to explain consumer behavior. These beliefs became widely accepted, and the SOR model also evolved into one of the most influential frameworks in environmental psychology (Yadav and Chakrabarti, 2022). The SOR model provides a concise, sophisticated framework that allows us to capture the unique environmental cues of live streaming and examine the internal state of consumers.

#### 2.2.1 S-social cues and media cues

As for the environmental cues, some scholars considered it as a holistic concept, as consumers simultaneously perceive all environmental elements and form an overall impression (Garaus, 2017). Others conducted detailed discussions on various elements of the environment. With the accumulation of some specific cues’ effects, scholars began to develop typologies and classification schemes, attempting to achieve some controllable influence through intentional design. As mentioned before, the key contextual cues in LSE are defined as social and media cues.

Achieving face-to-face interaction with streamers is the core advantage of LSE. Interactivity is a multi-dimensional, and complex concept. Generally, it’s measured in two different ways: feature-based vs. perception-based interactivity (Yang and Lee, 2017). Objective interactions obtained through experimental manipulation are feature-based, which focus on the media’s ability to create content. In contrast, the interaction measured by the scale is subjective and perceived. Whether and to what extent the media potential can be realized depends on the latter, which plays a more important role in shaping user behavior. In general, perceived interaction was divided into three dimensions: perceived control, responsiveness, and personalization (Wu, 2006). Mero (2018) proved that “live chat” can help e-retailers improve service quality. They defined it as two-way communication and called on academics to explore the role of other dimensions of interaction. Kang et al. (2021) advocated that, as a digital interactive platform, LSC naturally has the property of user control. Based on the above, we adopt responsiveness and personalization to represent the intensity and richness that users perceive from two-way communication with streamers.

The danmaku system in LSE facilitates interaction among community members. It’s considered as a more reliable information channel. Previous research has revealed peers’ influence in various contexts. In SNSs, peers’ sharing behaviors are just like the spread of viruses. The strength of its influence depends on the scale of friends (Zhu et al., 2016), individual-level tie strength, and group-level identification (Wang et al., 2012). On e-commerce platforms, peers usually involve a wider range of strangers. Peers’ influences typically stem from two forms of online social interaction: Word of Mouth (WOM) and Observational Learning (OL). WOM generally contains more detailed information, whereas OL only reveals behaviors (Cheung et al., 2014). Since behaviors speak louder than words, OL is generally considered more trustworthy. However, peers’ behaviors and opinions are usually expressed in the form of purchase quantity and post-purchase comments in traditional e-commerce, which belong to a lagging information exchange mode. In LSE, peers’ instant interaction alters the transmission mechanism of social influence. Through a systematic literature review, Xu et al. (2023) called for unpacking the power of viewer-viewer interaction in online communities.

Media cues are another stimulant. The unpredictability of live content heightens viewers’ experience of authenticity. Previous studies have investigated the influence of the authenticity of the core product and venue on the audience’s flow experience and recommendation intention in theater consumption (Aykol et al., 2017). Realness experience is a potentially important and interesting topic, yet its market performance has not been well understood (Ang et al., 2018). Furthermore, previous studies have demonstrated that vividness can improve consumers’ attitudes toward products. Fortin and Dholakia (2005) argued that technological development enable advertisers to leverage various characteristics of new media to enrich the interface and achieve better communication goals. Therefore, many platforms have endeavored to heighten the vividness of the interface and improve the overall attractiveness. E-retailers were eager to adopt innovative display tools to meet consumers’ sensory demands for product experience. Live streaming product displays with multiple media options may yield more positive results.

#### 2.2.2 O-pleasure, arousal, and perceived uncertainty

Some studies divide emotions into opposite valences, such as positive and negative emotions (Bellini et al., 2017). This overgeneralized classification fails to provide sufficient diagnostic value. Some studies have unearthed intriguing differences among subdivided emotions of the same valence (Cao and Sun, 2018). In this study, pleasure and arousal are considered as specific emotional states (Stoyanova et al., 2015; Xu et al., 2020a). Moreover, perceived uncertainty is adopted to measure cognitive state. Uncertainty about product quality and after-sales service are the two main reasons that prevent consumers from engaging in live streaming shopping (ii Media, 2020). Ma (2017) defined perceived uncertainty in logistics services as unexpected results and costs related to information asymmetry. Pavlou et al., (2005) argued that adverse selection and moral hazard existed in the relationship between buyers and sellers in e-commerce. The seller uncertainty stems from the buyer’s inability to fully evaluate the seller’s quality due to the seller’s previous misstatement of product characteristics (adverse selection) and fear of the seller’s opportunism (moral hazard) after the fact (Dimoka et al., 2012). The information system literature focuses on the seller’s uncertainty but ignores the product uncertainty. Apart from the lack of disclosure of the true attributes and future performance of the product caused by the seller’s subjective malice, there is also the possibility that the seller cannot fully describe the characteristics of the product or is unaware of all hidden problems. The description uncertainty or performance uncertainty caused by objective reasons is product uncertainty. The products involved in LSE are mainly experience products. The virtual environment exacerbates uncertainty by making it impossible for consumers to try and inspect the products in person. Dimoka et al. (2012) and Al-Adwan and Yaseen (2023) believe that the relationship between the seller and product uncertainty exhibits bidirectionality. Therefore, we conceptualize it as a whole construct.

#### 2.2.3 R-Urge to buy impulsively

Urge to buy impulsively is a specific state of mind, an experience in the desire for an object (Beatty and Ferrell, 1998). Previous studies have attempted to measure impulse buying through recall surveys or observations. This may lead to biased and inaccurate results (Bellini et al., 2017) because people will react in a social-expected way. Impulse buying is often treated as unwelcome, obsessive behavior, pathological addiction mania, or lack of self-control (Vohs et al., 2008). People tend to maintain the image of rationality. Chan et al. (2017) described the relationship between urge to buy impulsively and impulse buying as a knock-on reaction. Thus, we regard urge to buy impulsively a reasonable proxy of impulse buying.

## 3 Research model and hypothesis development

**Figure 1:**
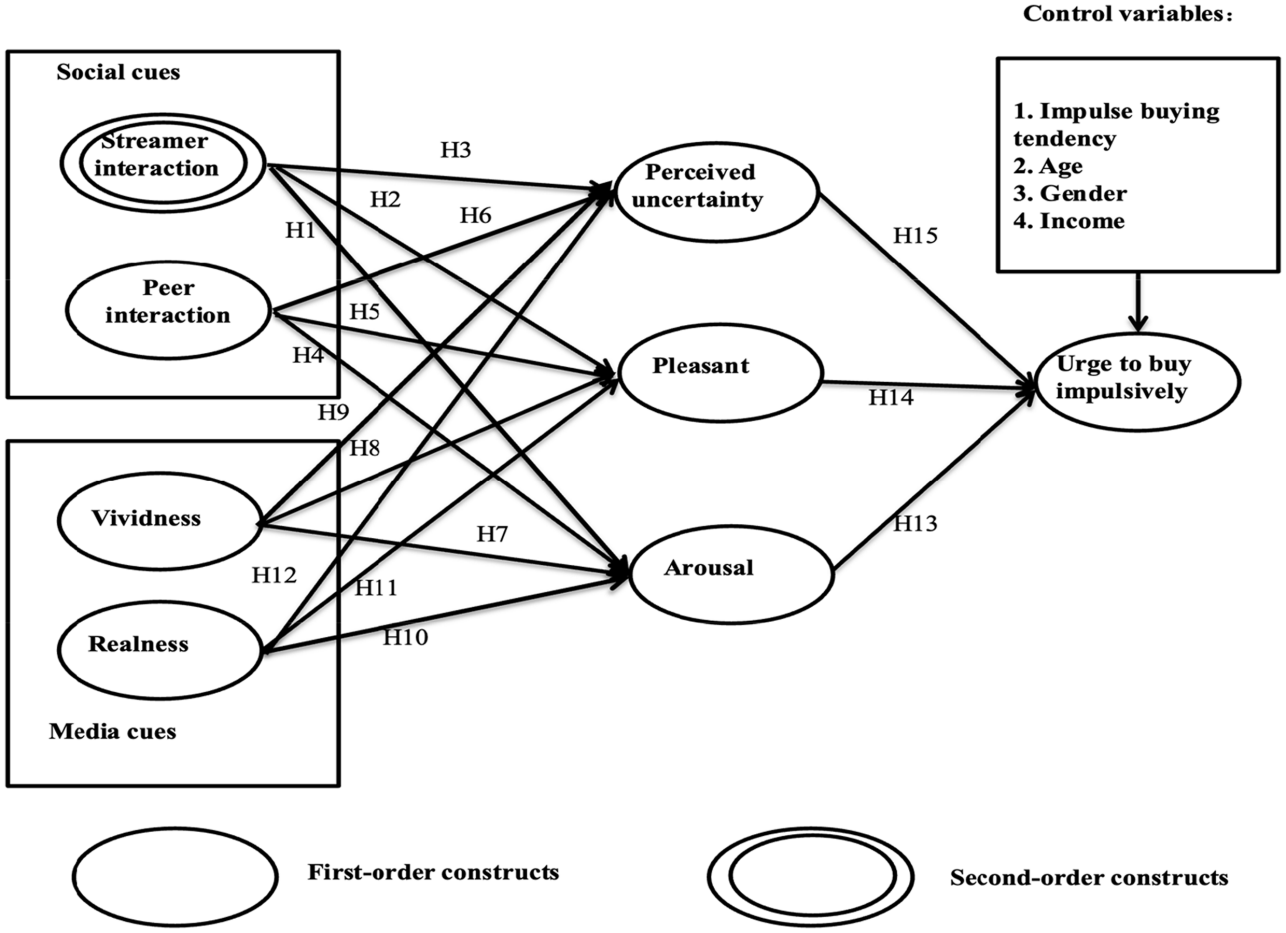
Research model.

**Figure 2:**
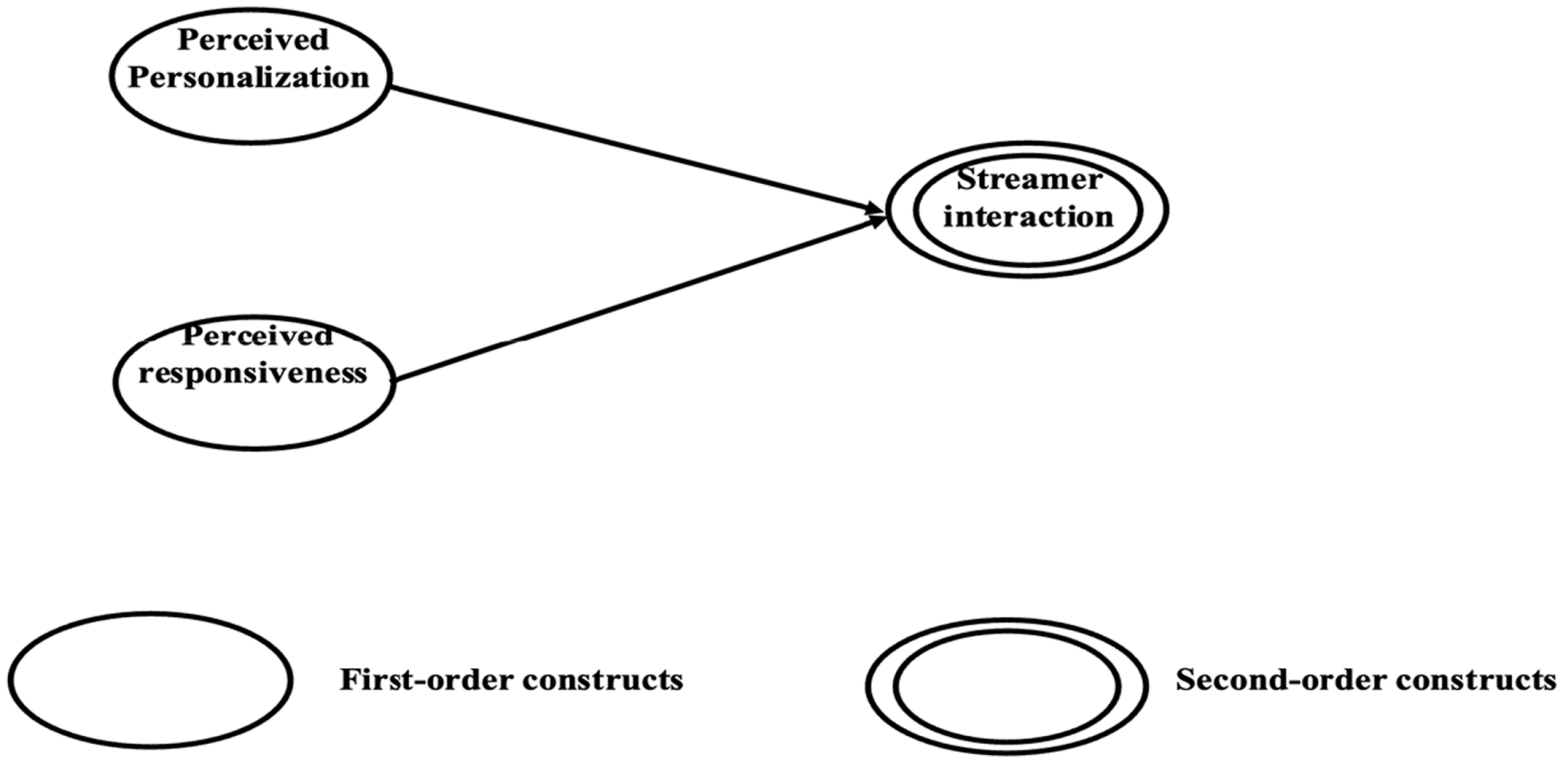
Second-order constructs.

Figure 1 depicts the conceptual model: social and media cues are posited as prerequisites for pleasure and arousal and will alleviate consumers’ perceived uncertainty. Subsequently, they will induce urge to buy impulsively. Streamer interaction is conceptualized as a formative second-order construct, which is created by perceived personalization and perceived responsiveness (as shown in Figure 2).

### 3.1 Effects of social cues

Perceived personalization and perceived responsiveness represent the intensity and richness of streamer interaction, respectively. Responsiveness indicates the probability and speed of the streamer’s response to questions, while individualization reflects the degree to which the streamer provides personalized communication for questions to meet the needs of users (Lee, 2005). In traditional e-commerce, consumers obtain product information through self-service searches and one-way reading. Searching for products, comparing alternatives, and evaluating information requires a lot of cognitive effort. In LSE, streamers can provide personalized suggestions for them (Sun et al., 2019). Consumers’ ability to evaluate products will be improved, and their sense of self-efficacy will be enhanced, which will make them feel happy. This deep interactive experience also enables consumers to participate in shaping the live content, thus elevating mundane shopping experiences to fashion. According to the Effectance theory, individuals have an internal psychological impulse to affect the environment (Wang et al., 2019), and when it’s successfully implemented, they will experience a positive feeling. Unlike the one-on-one interaction of traditional e-commerce, in an active studio, a streamer interacts with many customers (Wongkitrungrueng et al., 2020). Those who receive timely responses seem to successfully win this attention competition. This novel experience is likely to evoke excitement and enthusiasm among consumers. Thus, we propose:

Hypothesis 1 (H1): Streamer interaction has a positive effect on arousal.

Hypothesis 2 (H2): Streamer interaction has a positive effect on pleasure.

In s-commerce, Zhou et al. (2023) confirmed that interpersonal interaction with online vendors and recommenders helps to establish swift guanxi and initial trust. This interpersonal trust can translate into trust in the product (Wu and Huang, 2023). In the studio, consumers can consult streamers until they get satisfactory answers. High-quality and fruitful discussions serve to reinforce their relationship (Guan et al., 2020). Kang et al. (2021) also believe that personalized responses in s-commerce will make consumers feel cared for and respected, thus creating a strong sense of social identity and social presence, which is conducive to closer community ties. Huo et al. (2023) proposed that in rural tourism settings, interaction between residents and tourists can foster mutual understanding, form common beliefs, and build unity and trust. Thus, we propose:

Hypothesis 3 (H3): Streamer interaction has a negative effect on perceived uncertainty.

Peer interaction refers to the extent to which peers’ opinions and behaviors are visible through live streaming (Chen et al., 2011). In LSE, consumers with similar preferences gathered in a studio. To encourage them to stay in the studio until the last minute, streamers often offer special discounts in a countdown following the introduction. Peers adding products to shopping carts and processing orders can create a competitive atmosphere that may evoke emotional experiences. Wu and Gao (2019) believe that peer interaction in online tutoring helps to find common opinions and differences and co-construct new knowledge. The majority of consumers do not directly engage in live interaction but focus on the public screen (Chen et al., 2019). Consumers may get the information they want from peer inquiries, discover unique problems that they have not thought about, or increase the path of unexpected product discovery (Wang and Wu, 2019). These valuable interactions may contribute to heightened pleasure levels. Since consumers can find emotional resonance from similar views, obtain a sense of belonging and self-enhancement, and obtain the effect of vicarious learning from observation to acquire more product knowledge (Li et al., 2020). Zhao et al. (2019) believe that spiritual resonance and coordination with members of the community can bring great enjoyment. Thus, we propose:

Hypothesis 4 (H4): Peer interaction has a positive effect on arousal.

Hypothesis 5 (H5): Peer interaction has a positive effect on pleasure.

According to the Uncertainty Reduction theory, people in an uncertain environment may adopt proactive strategies, such as observing the behavior of similar others to achieve equilibrium (Knobloch, 2016). In the studio, peer entries, inquiries, thumbs up, comments, etc. are shown scrolling in real-time, providing opportunities for observation, making the transaction process more transparent, and creating a sense of social presence with others. Lu et al. (2016) believed that websites with high social interaction levels convey more information and enhance environmental transparency, thus restraining untrustworthy behaviors and making consumers feel more secure when making decisions. Leeraphong and Sukrat (2018) believe that perceived crowds will increase trust in merchants. Liu et al. (2023) also confirm that the transparency of virtual environment information can increase consumers’ trust in product safety and suppliers. Thus, we propose:

Hypothesis 6 (H6): Peer interaction has a negative effect on perceived uncertainty.

### 3.2 Effects of media cues

Vividness is defined as the richness of the media and the degree of sensory stimulation during the presentation of the product in live streaming (Sheng and Joginapelly, 2012). Arora et al. (2023) discovered that through the multi-sensory stimulation of vivid information in social media advertisements, users can enjoy additional happiness and generate empathy. In LSE, streamers walk around, zoom in, and explain various angles and details of the products. Consumers can obtain many non-verbal cues, such as exaggerated facial expressions, expressive body language, and vibrant tones. These visual and auditory cues may manipulate the viewer’s emotions. Yim et al. (2017) believed that media-driven vividness enriches consumers’ imaginative processes, allowing them to transcend the constraints of their physical environment, partake in the exploration of new products, and obtain adventure-like experiences. Hess et al. (2009) suggested that vividness fosters a friendlier, warmer environment. The rich sensory experience in LSE may satisfy consumers’ psychology of seeking entertainment. Thus, we propose:

Hypothesis 7 (H7): Vividness has a positive effect on arousal.

Hypothesis 8 (H8): Vividness has a positive effect on pleasure.

Vivid media information involves more human senses. In LSE, streamers can provide more abundant sensory cues. According to the cross-sensory compensation theory, the loss of other senses can be compensated by the visual and auditory senses (Mou et al., 2024). The vividness brought by multi-sensory channels can shape the experience of more specific simulated trial products (Huang and Chung, 2024), thereby reducing product uncertainty. Vividness can also help consumers bridge existing information and prior knowledge to form a composite picture and increase understanding of product quality (Hsu et al., 2024). Besides, in s-commerce, mental imagery vividness can enhance cognitive and affective social presence (Vazquez et al., 2023), which will provide opportunities for trust-building. Thus, we propose:

Hypothesis 9 (H9): Vividness has a negative effect on perceived uncertainty.

Realness denotes the degree to which products and shopping scenes are authentically reproduced in live streaming (Rubin, 1981). Consumer quest for authenticity. Live streaming makes them feel truly connected with the products. Augmented reality, as discovered by Hsu et al. (2024), imbues consumers with the sensation that products are just in front of their eyes, fostering instant gratification. The extraordinary experience brought by realness can create a flow state for theatergoers (Aykol et al., 2017), which is positively correlated with arousal (Sreejesh et al., 2020). The near-real product interaction facilitated by live streaming may enhance the intensity of positive emotions. Long and Tefertiller (2020) also believe that the more real information viewers get from live streaming, the more fun they will have. Thus, we propose:

Hypothesis 10 (H10): Realness has a positive effect on arousal.

Hypothesis 11 (H11): Realness has a positive effect on pleasure.

Pictures and videos can be edited afterward, so there is often a huge disparity between the “buyer show” and the “seller show.” LSE enables the viewer to connect with the scene in real-time, mitigating the loss of information in the process of multi-level transmission (Tong, 2017). Advanced display technology enhances consumers’ ability to capture and understand the products (Hsu et al., 2024). This direct interaction with the product can alleviate consumers’ concerns regarding information asymmetry. The spatial presence brought by authenticity may shorten the physical distance of consumers and increase their trust in sellers. Wolf (2020) also believes that realness plays a pivotal role in cultivating trust when using social media for product promotion. Thus, we propose:

Hypothesis 12 (H12): Realness has a negative effect on perceived uncertainty.

### 3.3 Effects of pleasure and arousal

Pleasure and arousal represent two positive emotional states. Pleasure denotes the degree of joyful and happiness that users feel from watching live streaming (Stoyanova et al., 2015; Chan et al., 2017), while arousal means the degree of excitement, stimulation, or activity users feel (Stoyanova et al., 2015). Urge to buy impulsively refers to a sudden, spontaneous desire to buy something (Beatty and Ferrell, 1998). Mattila and Wirtz (2008) believed that a high level of excitement would destroy self-control. Because positive emotions cause people to process information in a more heuristic way (Griskevicius et al., 2010), and to reward themselves more generously. They expect instant gratification to maintain them in a positive state (Floh and Madlberger, 2013). Dhurup (2014) claimed that beyond economic motives, the desire for pleasure often drives impulsive purchase. Destari et al. (2020) maintained that people with positive emotions are more willing to spend time browsing websites, thus increasing the chances of impulse buying. Thus, we propose:

Hypothesis 13 (H13): Arousal has a positive effect on urge to buy impulsively.

Hypothesis 14 (H14): Pleasure has a positive effect on urge to buy impulsively.

### 3.4 Effects of perceived uncertainty

Perceived uncertainty refers to the extent to which buyers cannot accurately predict the outcome of a transaction due to product or seller uncertainty (Dimoka et al., 2012; Chen and Huang, 2013). Impulse buying still involves thoughtful decision-making. Research on young Swedish shows that online fashion consumers strive for rationality even amidst emotional influence (Lidholm et al., 2017). Dholakia (2000) argues that individuals exposed to stimuli also generate cognitive responses, trying to identify any constraints in the environment. These constraints may lead to resistance or inhibition of impulsive behavior. In LSE, if consumers are unsure whether a product is suitable for them and whether the quality of the product is consistent with the description of the streamer, these concerns may hinder their urge to buy. Thus, we propose:

Hypothesis 15 (H15): Perceived uncertainty has a negative effect on urge to buy impulsively.

To control for any potential effects on urge to buy impulsively, we added impulse buying tendency, age, gender, and income level as control variables.

## 4 Data collection and empirical analysis

### 4.1 Measurement development

The questionnaire was developed by adapting scales that have been verified in previous studies with slight modifications tailored to the LSE context. For all measures, a five-point Likert scale was used with anchors ranging from strongly disagree “1” to strongly agree “5.” Since the survey was conducted in China, we used a forward-backward-translation approach to ensure consistency with the original scale (Sun et al., 2019). The finally formed measurement scales are shown in Appendix 1.

### 4.2 Data collection

This research launched an online survey through the “https://www.wjx.cn/,” inviting users who have participated in Taobao Live to support the survey. IP address restriction was set up to prevent duplicate responses. To ensure respondents have the same understanding of LSE, we introduced Taobao live at the beginning of the questionnaire and provided video links and screenshots with annotations for respondents in need. A total of 458 samples were received. According to pre-screening questions, abnormal response time, regular answers, and reverse-coded questions, 403 valid samples were screened out for further analysis. The sample size meets the criterion of Chin (1998).

Table 1 lists the demographic information of the respondents. The statistical results demonstrate that the sample is representative. Consistent with the research of Xu et al.’s (2020b) research, young and highly educated people are the dominant force in social commerce consumption. The gender distribution closely aligns with the data released by the Foresight Industry Research Institute (Chen, 2021).

**Table 1:** Demographic information of the respondents (*N* = 403).

Measure	Items	Frequency	Percentage
Gender	Male	141	34.99
	Female	262	65.01
Age	Below 18 years	3	0.74
	18 ~ 25 years	170	42.18
	26 ~ 30 years	27	6.70
	31 ~ 40 years	113	28.04
	41 ~ 50 years	82	20.35
	Above 50 years	8	1.99
Education	High school or below	12	2.98
	Vocational/technical school	27	6.70
	College student	222	55.09
	Graduate or above	142	35.24
Monthly income	Less than CNY 3000	148	36.72
	CNY 3000–4999	63	15.63
	CNY 5000–7999	98	24.32
	CNY 8000–10000	63	15.63
	More than CNY 10000	31	7.69

### 4.3 Data analysis and results

SPSS25.0 and SmartPLS3.2.8 (Ringle et al., 2015) were adopted for data analysis. PLS (partial least squares) is superior to multiple regression. It has a minimum requirement on sample size, can process non-normally distributed data, and deals with second-order formative variables (Sun et al., 2019). Streamer interaction is a formative higher-order construct, so its parameters are estimated by a repeated indicator approach and two-stage approach (Ringle et al., 2012). The repeated indicator approach is used to get the latent variable scores of two lower-order constructs, then these scores serve as manifest variables for the measurement of streamer interaction.

#### 4.3.1 Measurement model

##### 4.3.1.1 Common method bias and multicollinearity

According to Podsakoff et al. (2012), various procedural remedies were implemented to reduce Common Method Bias (CMB). For instance, ensuring respondents’ anonymity, randomizing the questions’ order, and positioning demographic inquiries at the end of the questionnaire. Finally, Harman’s single-factor test was conducted to address potential biases. SPSS25.0 was employed to perform principal component analysis. The number of the identified factors was more than 1, and the first factor accounted for 18.97% of the variance, lower than the threshold of 50% (Teo and Liu, 2007). Moreover, the variance inflation factors (VIF) values were calculated by SmartPLS to eliminate multicollinearity concerns. As illustrated in Table 2, VIF values ranged from 1.351 to 2.476, below the cutoff level of 5 (Hair et al., 2016).

**Table 2:** Weights, VIF, factor loadings, and cross-loadings.

Constructs	Items	VIF	Weights	Factor loadings and cross-loadings								
				PP	PR	PI	VI	RE	PU	AR	PL	UR
SI	PP	1.231	**0.612**									
	PR	1.231	**0.569**									
PP	PP1	1.695		**0.793**	0.345	0.132	0.160	0.291	−0.196	0.018	0.289	0.159
	PP2	1.738		**0.812**	0.380	0.100	0.098	0.319	−0.256	0.019	0.228	0.155
	PP3	2.086		**0.853**	0.324	0.015	0.078	0.24	−0.152	0.044	0.222	0.098
	PP4	1.623		**0.785**	0.356	0.033	0.077	0.261	−0.324	−0.019	0.204	0.07
PR	PR1	1.648		0.341	**0.793**	0.099	0.156	0.211	−0.214	0.102	0.227	0.117
	PR2	1.546		0.350	**0.764**	0.069	0.097	0.23	−0.170	0.106	0.166	0.095
	PR3	1.631		0.332	**0.785**	0.072	0.161	0.237	−0.199	0.087	0.238	0.139
	PR4	2.034		0.363	**0.856**	0.054	0.120	0.199	−0.148	0.110	0.155	0.085
PI	PI1	1.612		0.075	0.061	**0.847**	0.180	0.107	−0.064	0.268	0.256	0.234
	PI2	1.835		0.079	0.083	**0.815**	0.150	0.098	−0.083	0.161	0.165	0.159
	PI3	1.646		0.066	0.089	**0.852**	0.266	0.062	−0.026	0.268	0.271	0.211
VI	VI1	1.545		0.082	0.111	0.198	**0.784**	0.121	−0.118	0.210	0.257	0.102
	VI2	1.762		0.089	0.117	0.111	**0.771**	0.121	−0.075	0.115	0.182	0.044
	VI3	1.72		0.113	0.157	0.223	**0.817**	0.153	−0.099	0.205	0.270	0.157
	VI4	1.472		0.114	0.136	0.21	**0.781**	0.174	−0.128	0.229	0.259	0.135
RE	RE1	1.532		0.252	0.206	0.108	0.200	**0.786**	−0.243	0.049	0.235	0.161
	RE2	1.412		0.216	0.207	0.083	0.172	**0.751**	−0.254	0.094	0.172	0.145
	RE3	1.351		0.243	0.172	0.067	0.079	**0.742**	−0.269	0.024	0.216	0.202
	RE4	1.544		0.346	0.255	0.049	0.093	**0.744**	−0.171	0.029	0.175	0.130
PU	PU1	1.906		−0.241	−0.238	−0.055	−0.111	−0.303	**0.846**	−0.274	−0.208	−0.235
	PU2	2.476		−0.212	−0.103	0.039	−0.027	−0.261	**0.861**	−0.095	−0.079	−0.141
	PU3	1.902		−0.243	−0.164	−0.081	−0.164	−0.233	**0.812**	−0.196	−0.181	−0.168
	PU4	1.892		−0.252	−0.228	−0.100	−0.143	−0.25	**0.829**	−0.126	−0.207	−0.211
AR	AR1	1.621		−0.001	0.078	0.315	0.240	0.084	−0.171	**0.813**	0.298	0.263
	AR2	1.739		0.054	0.142	0.212	0.209	0.100	−0.195	**0.822**	0.304	0.305
	AR3	1.734		0.010	0.099	0.193	0.189	0.011	−0.210	**0.783**	0.243	0.231
	AR4	2.097		−0.006	0.088	0.182	0.149	−0.014	−0.102	**0.822**	0.219	0.170
PL	PL1	1.677		0.249	0.206	0.250	0.277	0.242	−0.184	0.293	**0.815**	0.316
	PL2	2.053		0.168	0.135	0.185	0.203	0.205	−0.082	0.166	**0.817**	0.179
	PL3	1.789		0.273	0.247	0.269	0.286	0.240	−0.216	0.291	**0.829**	0.277
	PL4	1.850		0.239	0.193	0.212	0.245	0.179	−0.175	0.321	**0.817**	0.293
UR	UR1	1.773		0.144	0.11	0.197	0.185	0.160	−0.172	0.274	0.321	**0.808**
	UR2	1.529		0.135	0.122	0.217	0.102	0.226	−0.236	0.270	0.265	**0.782**
	UR3	2.173		0.087	0.076	0.159	0.050	0.118	−0.144	0.195	0.183	**0.833**
	UR4	1.653		0.101	0.118	0.208	0.116	0.170	−0.180	0.237	0.279	**0.792**

PP, Perceived Personalization; PR, Perceived Responsiveness; PI, Peer interaction; VI, Vividness; RE, Realness; PU, Perceived uncertainty; AR, Arousal; PL, Pleasure; UR, Urge to buy impulsively (The following chart is the same). Streamer interaction is a formative variable; this column lists the factor weights (bold value) of PP and PR to Streamer interaction. In the factor loadings and cross-loadings part, the bold values are the factor loading values corresponding to the constructs and others are cross-loading values. All factor weights and factor loadings are significant (*p* < 0.001).

##### 4.3.1.2 Reliability and validity

Composite Reliability (CR) was used to examine the outer model’s reliability (Chin, 1998). Table 3 shows that the values of CR ranged from 0.842 to 0.904, both exceeding the required benchmark of 0.7 (Nunnally, 1978).

**Table 3:** CR, AVE, square root of AVEs, correlations between constructs and HTMT ratios.

Construct	CR	AVE	PP	PR	PI	VI	RE	PU	AR	PL	UR
PP	0.885	0.658	**0.811**	0.263	0.116	0.205	0.356	0.290	0.153	0.529	0.163
PR	0.877	0.640	0.433	**0.800**	0.102	0.155	0.381	0.227	0.238	0.336	0.264
PI	0.876	0.703	0.086	0.091	**0.838**	0.285	0.134	0.329	0.329	0.115	0.292
VI	0.868	0.622	0.127	0.167	0.244	**0.788**	0.228	0.366	0.286	0.155	0.170
RE	0.842	0.571	0.342	0.273	0.104	0.183	**0.756**	0.327	0.096	0.443	0.262
PU	0.904	0.701	−0.285	−0.228	−0.065	−0.137	−0.315	**0.837**	0.381	0.340	0.382
AR	0.884	0.656	0.020	0.127	0.288	0.250	0.065	−0.215	**0.810**	0.045	0.356
PL	0.891	0.672	0.290	0.245	0.285	0.314	0.267	−0.209	0.336	**0.820**	0.176
UR	0.880	0.646	0.148	0.135	0.246	0.147	0.214	−0.232	0.309	0.334	**0.804**

Diagonal: square root of AVEs report along diagonal in bold. Values below the diagonal: correlation between latent variables. Values above the diagonal: HTMT ratio.

Two criteria were adopted to test the convergent validity. The factor loading should exceed 0.7, and the average variance extracted (AVE) of each construct should exceed the variance due to the measurement error for that construct (Fornell and Larcker, 1981). The results in Table 2 indicate that all the values of factor loading exceed 0.736 and the results in Table 3 indicate that the AVE values ranged from 0.571 to 0.703, indicating that convergent validity is achieved.

Three methods were used to ensure discriminant validity. Firstly, the square roots of AVE should surpass the correlation coefficients of each construct (Fornell and Larcker, 1981) (shown in Table 3). Secondly, all factor loadings for each construct should exceed the cross-loadings (Chin, 1998) (shown in Table 2). Additionally, since streamer interaction is a second-order formative construct, traditional methods are inappropriate to evaluate its reliability and validity. The external weight of PR and PP are calculated to be 0.612 and 0.569, respectively, and significant, and the VIF values are less than 3.3 (as shown in Table 2), indicating the effectiveness of streamer interaction (Cenfetelli and Bassellier, 2009). Thirdly, in terms of discriminant validity, the Heterotrait-Monotrait (HTMT) ratio superior the Fornell-Larcker test (Henseler et al., 2015). As shown in Table 3, the HTMT values ranged from 0.045 to 0.529, all lower than 0.85, which verified the discriminant validity again (Voorhees et al., 2016).

#### 4.3.2 Structural model

**Figure 3:**
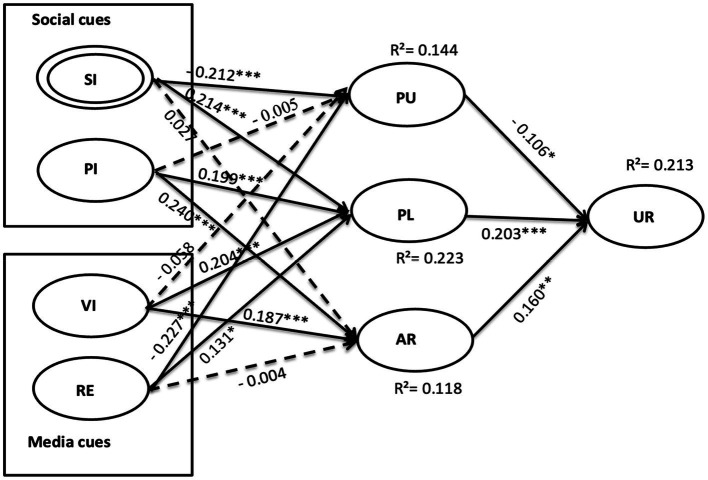
Model results. Numbers are standardized path coefficients. Dotted lines indicate insignificant paths. * *p* < 0.05, ** *p* < 0.01, *** *p* < 0.001.

With a competent measurement and acceptable levels of multicollinearity and CMB, the bootstrapping technique was implemented in SmartPLS to verify the structure model with 5,000 resamples test. The path coefficients (β) and significance (*p*-value), and explanatory power (R^2^) are illustrated in Figure 3 and Table 4.

**Table 4:** Hypotheses testing results.

Hypothesis	Path	Standardized coefficient	Sample mean (M)	Standard deviation (STDEV)	*p*-value	Support
H1	SI → AR	0.027	0.027	0.049	0.581	Not supported
H2	SI → PL	0.214***	0.214	0.048	0.000	Supported
H3	SI → PU	−0.212***	−0.213	0.045	0.000	Supported
H4	PI→AR	0.240***	0.242	0.046	0.000	Supported
H5	PI→PL	0.199***	0.201	0.048	0.000	Supported
H6	PI→PU	−0.005	−0.005	0.045	0.916	Not supported
H7	VI → AR	0.187***	0.190	0.052	0.000	Supported
H8	VI → PL	0.204***	0.206	0.046	0.000	Supported
H9	VI → PU	−0.058	−0.058	0.048	0.234	Not supported
H10	RE → AR	−0.004	−0.003	0.053	0.944	Not supported
H11	RE → PL	0.131*	0.133	0.051	0.010	Supported
H12	RE → PU	−0.227***	−0.231	0.046	0.000	Supported
H13	AR → UR	0.160**	0.160	0.055	0.003	Supported
H14	PL → UR	0.203***	0.203	0.050	0.000	Supported
H15	PU → UR	−0.106*	−0.107	0.046	0.022	Supported

*p < 0.05; **p < 0.01; ***p < 0.001.

Overall, 14.40% variance in perceived uncertainty, 11.8% variance in arousal, 22.3% variance in pleasure, and 21.3% variance in urge to buy impulsively were explained by this model. The standardized root means square residual (SRMSR) was 0.075, less than 0.08, indicating a good model fit (Hair et al., 2016).

## 5 Discussions and implications

### 5.1 Key findings and discussion

This study aims to reveal how social and media cues induce impulse buying in LSE. It proved that these impacts are mediated by consumers’ emotions and perception, and many interesting differences were discovered regarding the efficacy in the subdimensions of these cues.

As for social cues, contrary to expectations, streamer interaction cannot evoke an arousal experience. This may be because over-responding leads to core rigidity and learning myopia (Zhou et al., 2018). Perhaps streamers failed to provide unique content, as consumers become more experienced with watching live, they may expect more novel product knowledge and more entertaining engagement. It may also be that Taobao Live encourages shopkeepers to act as streamers, which, unlike those platforms characterized by the web celebrity model, cannot achieve the fan effect. Besides, unlike streamer interaction, peer interaction cannot effectively reduce perceived uncertainty. This is inconsistent with the discovery of Pandey et al. (2018), who argued that people’s sharing behavior on social media helps reduce risks and eliminate uncertainty. This may be because the text chat box allows large-scale participation, “noise” or the danmaku information updates too fast, limiting the depth of communication. Another possibility is that some sellers’ hiring of “water army” or fake fans undermines the credibility of peers’ information.

In terms of media cues, vividness cannot alleviate perceived uncertainty, although consumers can obtain more product information (Cai and Wohn, 2019) and have a closer psychological distance (Wu and Gao, 2019). This may be because previous research experimentally manipulated vividness (Vonkeman et al., 2017), producing information focused on the product itself. In LSE, streamers, products, viewers, multi-objects are involved in the studio (Zhang et al., 2022a). Vividness independent of the product may cause distraction and interfere with cognitive formation. Flashy elements, such as rockets or streamers’ overly flamboyant “performance” also tend to make the thematic argument get lost in the irrelevant background, which damages the signal transmission and hinders the formation of trust. Besides, realness cannot provoke excitement and passion as effectively as vividness. This is inconsistent with Verhagen et al., (2014) study, in which virtual mirrors caused product likability (including excitement and enthusiasm). One plausible explanation is that without the support of innovative technologies (such as AR), authenticity only met consumers’ most basic requirements for information quality. Alternatively, authenticity is a multi-dimensional concept encompassing streamer, product, and scene (Sun et al., 2022), where explicit authenticity at the visual level cannot inspire deeper psychological involvement.

Regarding the organism, emotions play a more important role in impulse buying than perception. This means that akin to social live streaming platforms, people expect relaxation and emotional enjoyment from their LSE consumption. Another possible explanation is that LSE predominantly involves experiential products, and complex shopping tasks encourage consumers to rely more on heuristic information processing patterns. What’s more, pleasure exerts a stronger impact than arousal, indicating that consumers tend to avoid overstimulation, which makes them feel burdened (Pelet et al., 2017).

### 5.2 Implications

#### 5.2.1 Theoretical implication

This study yields three primary theoretical implications.

Firstly, in the SOR framework, some general beliefs are often manipulated as stimuli, and developing stimulus classifications adapted to a specific context is a particularly intricate task. In LSE, the key contextual cues have changed (Xu et al., 2020a), including the content, theme, frequency of interaction (Xue et al., 2020), as well as the visual presentation of the transaction process (Chan et al., 2017). We conceptualize this deep interaction and the high quality of information provided by LSE into more concrete social and media cues. By integrating their subdimensions into an overall framework and comparing them, we revealed their different efficacy on the organism, thus providing a more precise explanation for impulse buying in LSE.

The second contribution is that, based on the emotion-cognitive framework, this study revealed the mental processes from external stimuli to behavioral responses.

Many studies emphasize affective factors as the drivers for impulse buying but deny the direct effect of cognitive factors (Zheng et al., 2019; Zhang et al., 2022a). Similarly, among the existing studies on live streaming business strategy, more research emphasizes the mediating role of emotional states, and focuses on community-triggered emotions (e.g., attachment, identification, and trust) while ignoring personal emotions (Xu et al., 2023). This research employs pleasure, arousal, and perceived uncertainty as mediators and demonstrates that two systems mediate the effects of environmental stimuli on impulse buying in parallel and reverse ways. While emotion exerts a more dominant role, quiet pleasure trumps excessive arousal.

Furthermore, previous studies have highlighted the importance of perceived uncertainty, but have not provided concrete answers. Traditional e-commerce emphasizes the role of institutional safeguards (e.g., credit rating, 7-day refund, etc.), while we revealed that exerting the influence of streamer interaction and utilizing the realness media property can also alleviate consumers’ uncertainty concerns.

#### 5.2.2 Practical implications

This study shed light on e-retailers and e-commerce platforms. By creating an attractive environment, they can improve sales performance and gain competitive advantages.

Firstly, given the best performance of streamer interaction in pleasing consumers and reducing uncertainty, designers should provide special mechanisms to facilitate this kind of communication. To adapt to the one-to-many interaction mode, a systematic text analysis function should be established, and the danmaku comments should be sorted according to the frequency, to get the most effective feedback on the core issues and alleviate the learning myopia. Besides, streamers should be creators of high-quality content, such as providing personalized recommendations based on product and viewer’s features. Furthermore, some gamified activities can challenge users’ inner enjoyment (Pelet et al., 2017). Therefore, streamers should increase such designs to encourage interactive engagement. Able e-retailers can harness the power of web celebrities to manipulate arousal levels.

In addition, peer interaction is the best way to arouse consumers, but its role in alleviating uncertainty needs to be improved. According to the social comparison theory, people often engage in upward comparisons, choosing a better reference person to improve their decision-making ability (Hu et al., 2019). Therefore, platforms and streamers should provide diverse rewards to encourage high-quality peer sharing. When the studio is overloaded with “noise,” it may prevent consumers from extracting valuable insights. Streamers should actively guide the content of live by setting up topic discussions. Platforms should weaken the display of non-task-related cues. For celebrity live streaming, driven by group identity, fans are more likely to be affected by herd consumption (Tseng and Lee, 2013). So, it’s beneficial to distinguish group identity through the strength of the relationship with the streamers (e.g., ‘Diamond Fans’). However, in the shopkeeper broadcast mode, the connection between community members is weak. Platforms should highlight helpful reviews through special fonts and delineate the identity of expert consumers via special markers to foster opportunities for upward imitation. Moreover, the platform should cooperate with the government regulatory policies, strengthen supervision, eliminate “water army,” and maintain a healthy ecology.

Thirdly, designers should meet viewers’ requirements for a multi-sensory experience. Virtual mirroring and augmented reality can be used to improve the vividness. Platforms should offer a “close” option to enable consumers to filter out flashy and irrelevant information. Moreover, in order to reduce perceived uncertainty, it is essential to reiterate the importance of explicit authenticity, but to create greater emotional value, e-retailers, and streamers should prioritize the hiring of mass models, and slang depictions (Wongkitrungrueng et al., 2020; Sun et al., 2022), as well as raw environments, to leverage the traditional culture attached to their products, and inspire an implicit authenticity.

Finally, for consumers, although impulse buying can serve as a form of “retail therapy” to reduce stress (Hsu et al., 2024), when making purchase decisions in LSE, it’s advisable to exclude the interference of information outside the product and identify false peers to avoid regret and trouble of returning.

In sum, through these efforts, viewers who are attracted by live streaming will generate a beneficial internal status, increasing their probability of buying.

## 6 Limitations and future research

Beyond these fruitful insights, some limitations should be bear in mind. Firstly, this study adopted a questionnaire to measure potential variables. Responders may be influenced by social desirability and subjectivity biases. Future research could consider more objective tools, such as electrodermal activity, pulse plethysmography, and electromyography. By triangulating these neurophysiological methods with experiments and investigations, more rigorous data can be obtained. Furthermore, the survey was conducted in China, with a dominant e-commerce platform (Taobao live) serving as the background. Due to the differences in the cultural and live streaming strategies across different platforms, the generalization of the conclusion should remain cautious. Finally, LSE is mainly applied to experience products, future research can take product type as a moderating variable to extend its application space in search products.^2^ Moreover, shopping experience and generational differences could also be considered as moderating factors to provide a deeper understanding of consumer behavior in LSE.

## Data availability statement

The original contributions presented in the study are included in the article/Supplementary material, further inquiries can be directed to the corresponding author.

## Ethics statement

Ethical approval was not required for the studies involving humans because according to local regulations and legislations, do not need to provide. The studies were conducted in accordance with the local legislation and institutional requirements. Written informed consent for participation was not required from the participants or the participants’ legal guardians/next of kin in accordance with the national legislation and institutional requirements because according to the national legislation and the institutional requirements, it is not required to write the informed consent. Written informed consent was not obtained from the individual(s) for the publication of any potentially identifiable images or data included in this article because the images are from the open, public website.

## Author contributions

YuX: Writing – review & editing, Writing – original draft, Visualization, Methodology, Investigation, Funding acquisition, Formal analysis, Data curation, Conceptualization. SC: Writing – review & editing, Writing – original draft, Supervision, Software, Project administration, Conceptualization. YiX: Writing – review & editing, Writing – original draft, Supervision, Project administration, Investigation, Funding acquisition, Data curation.

## Footnotes

^1^Products that can only be evaluated after trial, such as clothing, snacks, cosmetics.

^2^Products that can be evaluated by searching for information.
