# Supplementary material for: How social and media cues induce live streaming impulse buying? SOR model perspective
Source: Front Psychol. 2024 May 15;15:1379992. doi: 10.3389/fpsyg.2024.1379992 (PMC11133749; doi:10.3389/fpsyg.2024.1379992)
Supplement: Supplementary file 1 [file Data_Sheet_1.pdf]

Supplementary Material

How social and media cues induce live streaming impulse buying? SOR model perspective

Yu Xiang Xia, Seong Wook Chae*, Yi Cai Xiang

*** Correspondence:** Seong Wook Chae: swchae@hoseo.edu

Appendix 1. Questionnaire items.

Construct	Items	Reference
Streamer interaction	PP1: The streamer provides tailored answers to my questions.<br>PP2: The streamer recommends products suitable for me to match my personal needs.<br>PP3: The streamer makes me feel that I am a unique customer.<br>PP4: The streamer can focus on my needs for products or services.	Adapted from Lee (2005) and Xue et al. (2020)
	PR1: The streamer is ready and willing to respond to my needs.<br>PR2: The streamer can answer my questions and requests in time.<br>PR3: The streamer is very happy to communicate with me.<br>PR4: The streamer can't provide relevant information for my inquiry in time.*	Adapted from Xue et al. (2020) and Yang et al. (2020)
Peer interaction	PI1: In the live streaming studio, I can view other buyers' actions (e.g., adding products to the shopping cart; processing orders, or making payments) in real-time.<br>PI2: In the live streaming studio, I can view inquiries asked by other buyers and the feedback in real time.	Adapted from Wang and Wu (2019) and

Construct	Items	Reference
	PI3: In the live streaming studio, I can view other buyers' Danmaku comments in real time.	Wang et al. (2019)
Vividness	VI1: The product information contained in the live streaming stimulates my senses (e.g., I can observe the body language and expression of the streamer, and hear the voice and tone of the streamer).<br>VI2: Live streaming offers rich media information (e.g., Videos, enlarged pictures, comic emojis, etc.).<br>VI3: In the live streaming studio, I can acquire product information from different sensory channels.<br>VI4: The product demonstration in the live streaming is lively.	Adapted from Sheng and Joginapelly (2012) and Vonkeman et al. (2017)
Realness	RE1: Live streaming presents the real situation of the product.<br>RE2: Live streaming lets me see how others are using the product.<br>RE3: Live streaming does not show the real situation of the products.*<br>RE4: Live streaming lets me see what happens in other places as if I were really there.	Adapted from Rubin (1981)
Perceived uncertainty	PU1: I feel that purchasing products from this live streaming studio involves a high degree of uncertainty.<br>PU2: I feel the uncertainty associated with purchasing products from this live streaming studio is high.<br>PU3: I am exposed to many transaction uncertainties if I purchase products from this live streaming studio.<br>PU4: There is a high degree of product uncertainty (e. g. the product I receive may not be exactly what I want) when purchasing products from this live streaming studio.	Adapted from Zhang et al. (2019)

Construct	Items	Reference
Pleasure	PL1: I feel joyful when watching the live streaming.<br>PL2: I feel pleasure when watching the live streaming.<br>PL3: I feel happy when watching the live streaming.<br>PL4: I feel gratified when watching the live streaming.	Adapted from Huang et al. (2017)
Arousal	AR1: I feel enthusiastic when watching the live streaming.<br>AR2: I feel exhilarated when watching the live streaming.<br>AR3: I feel energized when watching the live streaming.<br>AR4: I feel excited when watching the live streaming.	Adapted from Xu et al. (2020b)
Urge to buy impulsively	UR1: As I watched this live streaming, I experienced a sudden urge to buy things I had not planned to purchase.<br>UR2: As I watched this live streaming, I wanted to buy something even though it's not on my shopping list.<br>UR3: As I watched this live streaming, I felt a sudden urge to buy something.<br>UR4: As I watched this live streaming, I was inclined to purchase things outside my specific shopping goal.	Adapted from Beatty and Ferrell (1998) and Parboteeah et al. (2016)

Note: *Reverse coded.

References

Beatty, S. E., and Ferrell, M. E. (1998). Impulse buying: modeling its precursors. J. Retail. 74, 169–191. doi: 10.1016/S0022-4359(99)80092-X

Huang, M., Ali, R., and Liao, J. (2017). The effect of user experience in online games on word of mouth: a pleasure-arousal-dominance (PAD) model perspective. Comput. Hum. Behav. 75, 329–338. doi: 10.1016/j.chb.2017.05.015

Lee, T. (2005). The impact of perceptions of interactivity on customer trust and transaction intentions in mobile commerce. J. Electron. Commer. Res. 6, 165–180.

Parboteeah, D. V., Taylor, D. C., and Barber, N. A. (2016). Exploring impulse purchasing of wine in the online environment. J. Wine Res. 27, 322–339. doi: 10.1080/09571264.2016.1204597

Rubin, A. M. (1981). An examination of television viewing motivations. Commun. Res. 8, 141–165. doi: 10.1177/009365028100800201

Sheng, H., and Joginapelly, T. (2012). Effects of web atmospheric cues on users' emotional responses in e-commerce. AIS Trans. Hum-Comput. Int. 4, 1–24. doi: 10.17705/1thci.00036

Vonkeman, C., Verhagen, T., and Van Dolen, W. (2017). Role of local presence in online impulse buying. Inform. Manage. 54, 1038–1048. doi: 10.1016/j.im.2017.02.008

Wang, K., Pan, Z., Lu, Y., and Gupta, S. (2019). What motives users to participate in danmu on live streaming platforms? The impact of technical environment and effectance. Data Inf. Manage. 3, 117–134. doi: 10.2478/dim-2019-0013

Wang, X., and Wu, D. (2019). "Understanding user engagement mechanisms on a live streaming platform" in HCI in business, government and organizations. Information systems and analytics. eds. F. F.-H. Nah and K. Siau (Switzerland: Springer International Publishing), 266–275.

Xu, H., Zhang, K. Z. K., and Zhao, S. J. (2020b). A dual systems model of online impulse buying. Ind. Manage. Data Syst. 120, 845–861. doi: 10.1108/IMDS-04-2019-0214

Xue, J., Liang, X., Xie, T., and Wang, H. (2020). See now, act now: how to interact with customers to enhance social commerce engagement? Inform. Manage. 57:103324. doi: 10.1016/j.im.2020.103324

Yang, K., Kim, H. M., and Zimmerman, J. (2020). Emotional branding on fashion brand websites: harnessing the pleasure-arousal-dominance (P-A-D) model. J. Fashion Mark. Manage. 24, 555–570. doi: 10.1108/JFMM-03-2019-0055

Zhang, M., Qin, F., Wang, G. A., and Luo, C. (2019). The impact of live video streaming on online purchase intention-work. Serv. Ind. J. 40, 656–681. doi: 10.1080/02642069.2019.1576642
